# Supplementary material for: FEABAS: A Stitching and Alignment Tool for Serial EM Data
Source: bioRxiv. 2026 Jun 10:2026.06.07.730510. Preprint. [Version 1] doi: 10.64898/2026.06.07.730510 (PMC13277924; doi:10.64898/2026.06.07.730510)
Supplement: Supplementary file 1 [file NIHPP2026.06.07.730510v1-supplement-1.pdf]

bioRxiv preprint doi: https://doi.org/10.64898/2026.06.07.730510; this version posted June 10, 2026. The copyright holder for this preprint (which was not certified by peer review) is the author/funder, who has granted bioRxiv a license to display the preprint in perpetuity. It is made available under a CC-BY 4.0 International license.

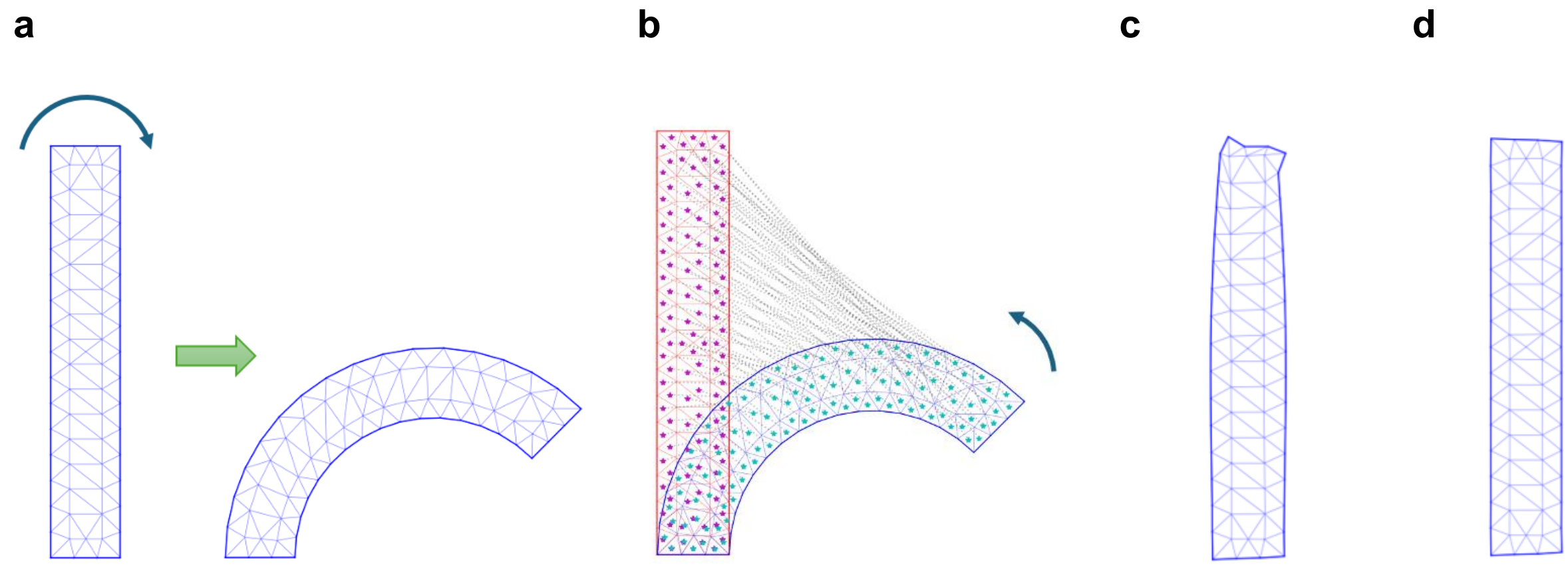

Supplementary Figure 1: **Demonstration of Rotation-Variant (Linear) vs. Rotation-Invariant (hyperelastic) Elasticity Models. (a)** Geometric setup of the elastic stress test. An undeformed rectangular reference mesh (left) is manually bent into an arch-like geometry (right), introducing a severe, non-uniform rotation gradient from one end to the other end of the structure. **(b)** Landmark constraint configuration. To reverse the deformation, landmark correspondences (asterisks) are established at the centroid of every triangular element, mapping points from the deformed target mesh (blue) back to their original coordinates in the undeformed reference configuration (red). **(c)** Optimization result using a standard linear elasticity model. Elements at the highly rotated end (top) exhibit severe non-physical distortions, due to the rotation-variant nature of the model. **(d)** Optimization result using the nonlinear St. Venant-Kirchhoff (SVK) hyperelastic model. By accounting for finite strains, the SVK model accommodates the large rotation without severe element distortions.

bioRxiv preprint doi: https://doi.org/10.64898/2026.06.07.730510; this version posted June 10, 2026. The copyright holder for this preprint (which was not certified by peer review) is the author/funder, who has granted bioRxiv a license to display the preprint in perpetuity. It is made available under a CC-BY 4.0 International license.

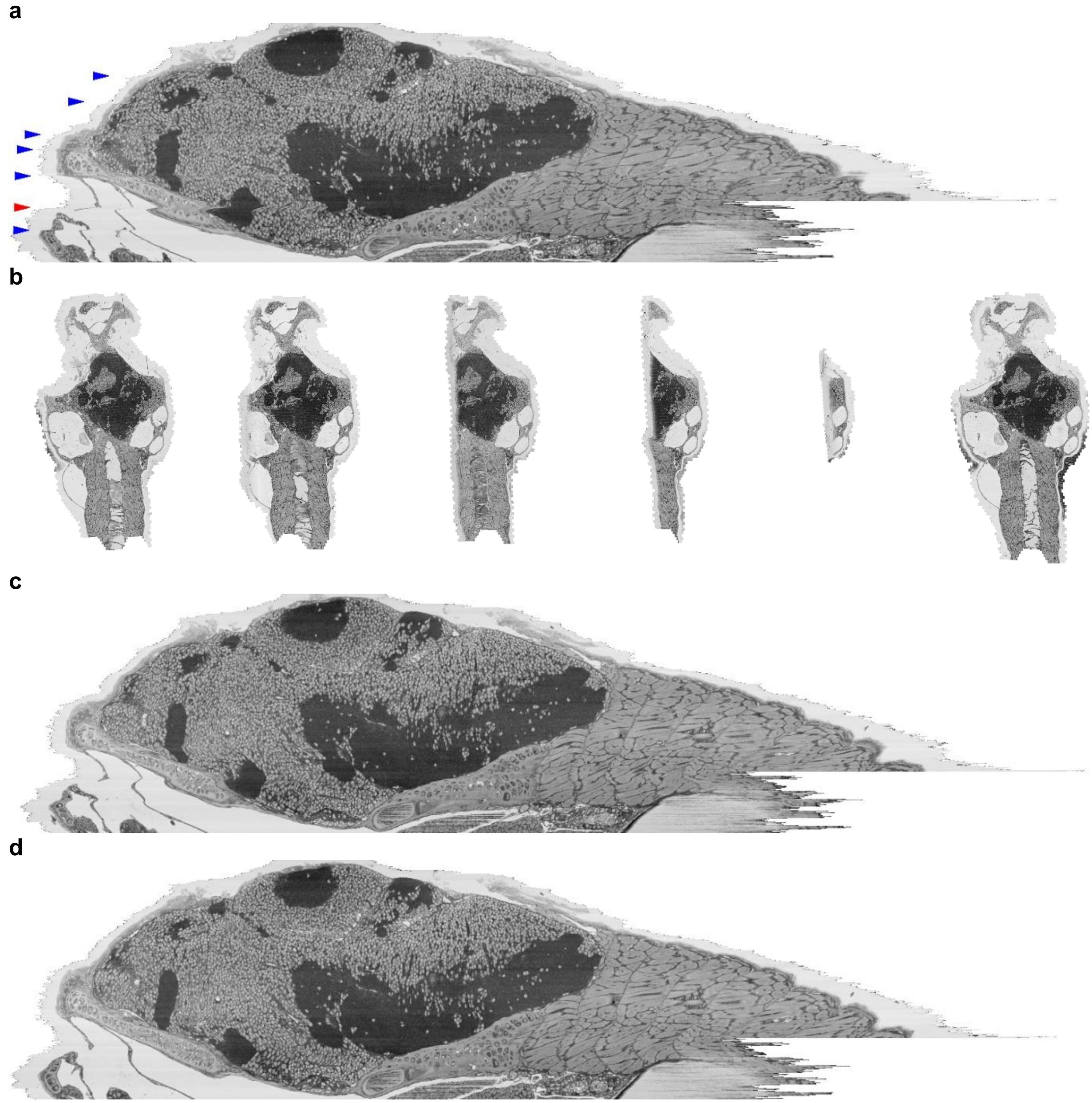

Supplementary Figure 2: **Mitigation of Volumetric Z-Drift**. **(a)** Virtual xz cross-section of the zebrafish dataset processed with default mesh stiffness. Significant axial drift is visible (arrows). The red arrow denotes the onset of the most severe discontinuity. **(b)** Serial thumbnails of the sections corresponding to the red arrow in a. The sequence illustrates a gradual transition from complete sections to partial fragments and back to complete sections, a hallmark of diamond knife replacement. **(c)** The same xz view as in a, processed with the mesh stiffness reduced to 0.05x the default value. The reduction in mechanical resistance allows the solver to better accommodate the geometric step-change, partially attenuating the observed drift. **(d)** xz reconstruction following three rounds of "annealing." In this process, the relaxed mesh state from each iteration is defined as the new resting position for the subsequent solve. This iterative realignment effectively eliminates the remaining drift.
